# Genome-wide association research on the reproductive traits of Qianhua Mutton Merino sheep

**DOI:** 10.5713/ab.23.0365

**Published:** 2024-04-01

**Authors:** Jiarong Li, Limin Sun, Jiazhi Sun, Huaizhi Jiang

**Affiliations:** 1 College of Animal Science and Technology, Jilin Agricultural University, Changchun, 130000, China; 2 Institute of Animal Husbandry and Veterinary, Jilin Academy of Agricultural Sciences (Northeast Agricultural Research Center of China), Changchun, 130000, China; 3 Anhua Agricultural Insurance Co., Ltd. Changchun Central Branch, Changchun, 130000, China

**Keywords:** Genome Analysis, Reproductive Traits, Sheep Breeds

## Abstract

**Objective:**

Qianhua Mutton Merino sheep is a new breed of meat wool sheep cultivated independently in China. In 2018, it was approved by the state and brought into the national list of livestock and poultry genetic resources. Qianhua Mutton Merino sheep have the common characteristics of typical meat livestock varieties with rapid growth and development in the early stage and high meat production performance. The objective of this research is to investigate the Genome-wide association of the reproductive traits of Qianhua Mutton Merino sheep.

**Methods:**

Qianhua Mutton Merino sheep from the breeding core group were selected as the research object, genome-wide association analysis was conducted on genes associated with the reproductive traits (singleton or twins, birth weight, age [in days] for sexual maturity, weaning weight, and daily gain from birth to weaning) of Qianhua mutton merino.

**Results:**

Our study findings showed that 151 loci of single-nucleotide polymorphisms (SNPs) were detected, among which 3 SNPs related to birth weight and weaning weight occupied a significant portion of the wide genome. The candidate genes preliminarily obtained were *SYNE1*, *SLC12A4*, *BMP2K*, *CAMK2D*, *IMMP2L*, *DMD*, and *BCL2*.

**Conclusion:**

We found 151 SNP loci for five traits related to reproduction (including singleton or twins, birth weight, age [in days] at sexual maturity, weaning weight, and daily weight gain from birth to weaning). The functions of these candidate genes were mainly enriched in nucleotide metabolism, metal ion binding, oxytocin signaling pathway, and neurotrophin signaling pathway.

## INTRODUCTION

Qianhua mutton merino is a new fine-wool sheep breed developed for dual purposes of mutton and wool and has gained independent intellectual property in China. This breed was given national authorization in China in 2018. It was bred by crossing South African mutton merino (as a male parent) with Northeast fine-wool sheep (as a female parent). This sheep lives in integrated regions of cultivation and grazing and has demonstrated strong adaptability, high early growth rate, great body weight, excellent meat quality, and high meat yield. Moreover, its lambing rate could reach more than 130%, and its coat is covered by homogeneous fine wool with 66 counts as the main form. This breed is an excellent source of species for the development of the Chinese mutton sheep industry.

Reproductive traits of sheep are complex economic traits modulated by numerous genes, and evidence for the major genes associated with the reproductive traits remains unclear. A genome-wide map of the livestock and poultry is available, and single-nucleotide polymorphism (SNP) chips with different densities have been developed and promoted. In addition to these phenomena, genome-wide association analysis (GWAS) has been performed to detect the critical candidate genes associated with the complex traits of livestock and poultry. Thus, it has become important to identify the critical candidate genes associated with key economic traits of livestock and poultry. Considering the cost and yield of sequencing, we applied sequencing-based methods for amplifying fragments of specific loci (specific-loci amplified fragment sequencing [SLAF-seq]) of pigs [1], which was also used for the genetic difference analysis of the ewe population of Qianhua Mutton Merino. The SLAF-seq technology was employed in this study to develop molecular markers using 200 blood samples from foreflower Merino sheep. By combining the original data obtained through measurement and calculation (Supplementary Table S1), whole genome molecular markers were acquired. This study also compared the differences in the genotype frequency of genome-wide SNP loci among treatment groups. The candidate genes were ascertained on the basis of physical locations of the SNP loci, and the selected candidate genes were subsequently subjected to data mining to make annotations for their molecular functions and biological processes. These results further provided guidance for the breeding and production of Qianhua mutton merino.

## MATERIALS AND METHODS

### Ethical statements

We conducted this study in compliance with the principles of the Laboratory Animal Center of Jilin Agricultural University (No. 2021 10 20 001).

### Analyzed indicators and methods

i) Birth weight and weaning weight of animals.

ii) Age (in days) at sexual maturity: This refers to the age (in days) when the ewes were subjected to the first successful mating.

iii) Average daily weight gain from birth to weaning = (weaning weight – birth weight)/(time for analysis of weaning weight − time for analysis of birth weight)

### Samples collection and DNA extraction

Experimental animals were selected from animals of the breeding central group of Qianhua mutton merino fed by Qiananzhihua Sheep Breeding Limited Company in Jilin Province. Briefly, 200 ewes that were born during January 10 to 13, 2018, were selected at random. The selected ewes were in good physical condition, good appetite and good health. All the ewes were subjected to house feeding. Upon weaning of the ewes (at the age of 90 days), the daily ration was designed according to the standards of National Research Council (NRC) (2007). The ewes were injected with various vaccines regularly, and insects on the ewes were expelled in a timely manner. Whole-blood samples were collected in ethylene diamine tetraacetic acid (EDTA) sampling tubes preplaced with an anticoagulant and were stored at −80°C for the subsequent DNA extraction. Genomic DNA from the whole blood was extracted using Genomic DNA Purifcation Kit (Termo Fisher Scientifc Inc., Waltham, MA, USA). Te concentration and purity of DNA were measured by Nano-Drop 2000 spectrophotometer (Termo Fisher Scientifc Inc., USA) and stored at −20°C for whole-genome sequencing.

### Library construction and whole genome sequencing

The purified polymerase chain reaction products were exported from the library for subsequent library quality inspection and computer-based sequencing. After quality inspection, the qualified library was sequenced using the Illumina platform. To ensure the quality of the bioinformatics analysis, raw reads were filtered to obtain clean reads for subsequent information analysis. The major steps in data filtration are as follows: i) reads with adapters are removed, ii) reads with N content above 10% are filtered, and iii) reads with over 50% of bases (whose quality value was below 10) are eliminated.

To explore models that fit the analysis of resource groups in this study, association analysis was performed using three software programs, namely, TASSEL [2], factored spectrally transformed linear mixed models (FaST-LMM) [3], and EMMAX [4], using data from developed high-density SNP molecular markers. Specifically, equations for the generalized linear model (GLM) and mixed linear model (MLM) of TASSEL software are as follows:


GLM: y=Xα+Qβ+e MLM: y=Xα+Qβ+Kμ+e

### Data processing software and databases

The software used for data analysis was as follows: TASSEL 5.0 (https://www.maizegenetics.net/tassel), FaST-LMM (https://www.microsoft.com/en-us/download/confirmation.aspx?id=52588), EMMAX (https://csg.sph.umich.edu//kang/emmax/download/index.html), R language (https://www.r-project.org/), Admixture software, EIGENSOFT 5.0 (https://www.hsph.harvard.edu/alkes-price/software/): Burrows-Wheeler aligner (BWA) software, (https://sourceforge.net/projects/bio-bwa/files/bwa-0.7.15.tar.bz2/download?use_mirror=netix&download=&failedmirror=nchc.dl.sourceforge.net). The databases used for data analysis is as follows: NCBI: https://www.ncbi.nlm.nih.gov/, Genome of sheep Ovis_aries_v1.0: hftp://ftp.ncbi.nlm.nih.gov/genomes/all/GCA/002/742/125/GCA_002742125.1_Oar_rambouillet_v1.0, DAVID website for annotation of online functions: https://david.ncifcrf.gov/

### Data processing and statistical analysis

Average daily weight gain of experimental ewes from birth to weaning was calculated using data on birth weight and weaning weight provided by Qiananzhihua Sheep Breeding Company Limited. Birth weight data were obtained from records of Qianhua mutton merino production in 2018, whereas weaning weight data were obtained through measurements during the process of practical production performance analysis. Statistical analysis was performed using SPSS 25.0 for data on birth weight, weaning weight, and daily weight gain from birth to weaning. Concomitantly, frequency distribution histograms were plotted for these three traits relevant to mutton quality.

### Computational analysis of linkage disequilibrium

Linkage disequilibrium (LD) of two loci occurs when the probability for simultaneous occurrence of a particular allele at one locus and another allele in another locus is greater than that for simultaneous occurrence of two alleles due to random distribution within a group. Conceptually, LD is related neither to chromosome nor to linkage. However, within a chromosome, the stronger the LD at two loci, the more compact the linkage. LD analysis could yield the smallest genetic unit of a species.

### Annotation and enrichment analysis

The OvineSNPs50 BeadChip genome location annotation files were used to locate the SNP position in the latest version of the genome sequencing tool (Ovis_aries_v1.0). The Ovisaries Annotation Release100 tool in NCBI was used to identify neighboring genes of sheep corresponding to the SNPs that showed significant association. Finally, the quantitative trait locus database was used to compare the significantly associated SNPs and the known quantitative trait loci (QTL) of sheep and verify the obtained data. Gene ontology (GO) is an integrated database that provides information on gene functions and classifies genes based on their essential functions, thus describing the functions of genes and proteins. Kyoto encyclopedia of genes and genomes (KEGG) is an integrated database containing information on genome, chemistry, and system function. The local platform of gene set enrichment analysis (GSEA) tools (https://www.gsea-msigdb.org/gsea/index.jsp) was used to perform enrichment analysis on the molecular function, biological process, and cellular components of genes. It can also select genes relevant to the traits of sheep growth and development according to the results of enrichment analysis.

## RESULTS

### Evaluation of sequencing quality value distribution

The sheep genome (Ovis_aries_v1.0) was selected as the reference genome in the electronic enzymolysis prediction. Finally, Hpy166II+EcoRV-HF was used for enzymolysis. A total of 453,722 SLAF markers, including 366,094 polymorphic SLAF markers, were obtained through bioinformatics analysis. A total of 6,157,562 population SNPs were obtained, which were filtered based on a minor allele frequency of above 0.05 and integrity of above 0.8. The SNPs obtained after filtration were used for evolutionary analysis.

### Distribution of single-nucleotide polymorphisms in chromosome

**Figure 1:**
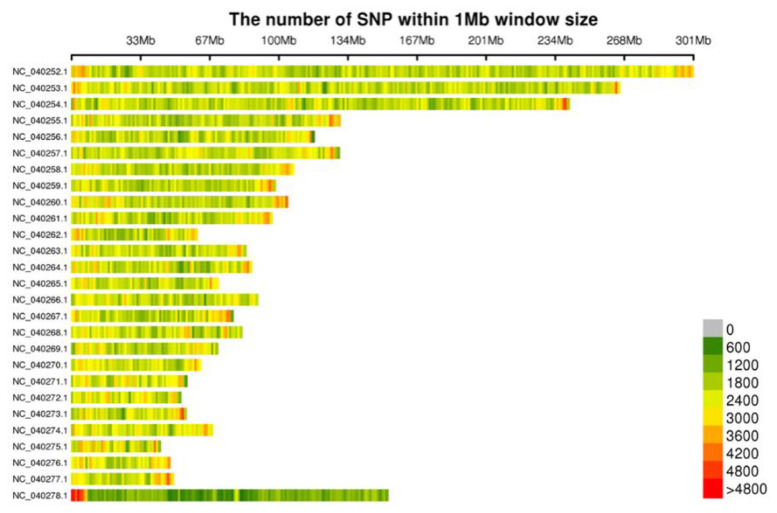
Distribution of single-nucleotide polymorphisms on chromosomes. Abscissa refers to the length of the chromosome. Each band represents a chromosome. The genome is divided according to the size of 1 Mb. The more SNP markers in each window, the darker the color, whereas the fewer SNP markers, the lighter the color. The darker area in the figure represents the area where the SNP markers are concentrated. SNP, single-nucleotide polymorphism.

The development of SNP markers was based on the sheep genome (Ovis_aries_v1.0) as the reference genome. BWA software was used for mapping the sequencing reads to the reference genome, and two methods (GATK [5] and samtools [6]) were used to develop the SNPs. The intersection of the SNP markers obtained using the two methods was considered the final reliable database of SNP markers. A total of 6,157,562 population SNPs were obtained. A diagram of the SNP distribution on the chromosome was plotted (Figure 1).

### Results of genome-wide association analysis for each trait

The Bonferroni method was used to determine the significant critical values used as a reference for the GWAS. At p<0.05/N or p<0.01/N, wherein N is the number of SNPs obtained during detection, the SNP became significant or extremely significant at the genome-wide level. Supplementary Table S2 lists information about chromosomes and the significant threshold values at the chromosome and genome-wide levels.

The software programs TASSEL, FaST-LMM, and EMMAX were used to perform GWAS, on the basis of GLM and MLM, for traits of singleton or twins, birth weight, age (in days) at sexual maturity, weaning weight, and daily weight gain from birth to weaning in the Qianhua mutton merino group comprising 200 ewes. After a comprehensive evaluation of the results of multiple models, the genome-wide analysis results obtained using EMMAX software were selected. As suggested from the GWAS results, 151 SNPs were significantly correlated (at the chromosome level) with five traits (namely, singleton or twins, birth weight, age [in days] at sexual maturity, weaning weight, and daily weight gain from birth to weaning) of the ewes of Qianhua mutton merino. Among them, three SNPs were significantly correlated (at the genome-wide level) with these five traits and were associated with birth weight and weaning weight.

**Figure 2:**
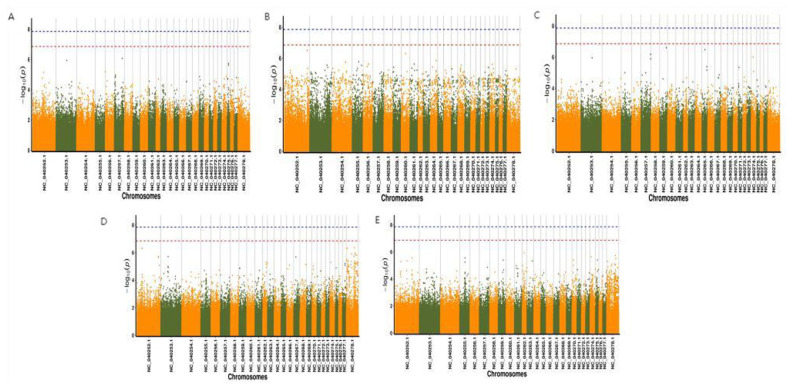
Results of genome-wide association analysis for each trait. (A) Manhattan plots of the genome-wide association analysis of singleton and twin traits in Qianhua mutton merino; (B) Manhattan plot of the genome-wide association analysis of birth weight in Qianhua mutton merino; (C) Manhattan plot of the genome-wide association analysis of age at sexual maturity in Qianhua mutton merino; (D) Manhattan plot of the genome-wide association analysis of weaning weight in Qianhua mutton merino; (E) Manhattan plot of the genome-wide association analysis of daily weight gain before weaning in Qianhua mutton merino. The abscissa represents the position of the chromosome, and the ordinate represents the p-value (−log10(p)), taking the negative logarithm based on 10. The scattered dots (or lines) on the graph represent the −log10(p) corresponding to each SNP site). The blue horizontal line represents 0.01/718040 = 1.39E-08, which means −log10(0.01/718040) = 7.86; the red horizontal line represents 0.1/718040 = 1.39E-07, which means −log10(0.1/718040) = 6.86.

In detail, 14 loci of SNPs were significantly correlated with the traits of singleton or twins, and all the loci were significant at the chromosome level. Among these loci, four were located on chromosome 6 and two were located on chromosome 21, while the others were located on chromosomes 2, 5, 11, 12, 20, 24, 25, and 26, respectively (Supplementary Table S3). Figure 2a illustrates the Manhattan plots of the GWAS.

Twenty-one loci of SNPs were significantly correlated with the birth weight trait, and all these loci were significant at the chromosome level. Among these loci, two demonstrated significances at the genome-wide level (given 0.5/718040 = 6.96E-07, i.e., −log10(0.5/718040) = 6.16), which are located on chromosomes 8 and 14. Among the other 19 SNP loci that exhibited significance at the chromosome level, two were located on chromosomes 2, 7, 16, and 20; the other loci were located on chromosomes 1, 4, 5, 6, 9, 11, 17, 23, 25, and 27 (Supplementary Table S4). Figure 2b shows the Manhattan plots of the GWAS.

Seventy-four loci of SNPs were significantly correlated with the trait of age (in days) at sexual maturity, and all the loci were significant at the chromosome level. Among these loci, 9 were located on chromosome 24, and 8 were located on chromosomes 17 and 22. Additionally, 6 loci were located on chromosome 5; 5 loci were located on chromosome 4; 4 loci were located on chromosome 6; 3 loci were located on chromosomes 7, 20, 25, and 27; 2 loci were located on chromosomes 1, 8, 10, 11, 13, 14, and 26; and the other loci were located on chromosomes 2, 3, 12, 15, 16, 19, and 23. Additionally, a locus of the SNPs was located in unknown chromosomes (Supplementary Table S5). Figure 2C shows the Manhattan plots of the GWAS of the trait of age (in days) at sexual maturity.

Twenty-one loci of SNPs were significantly correlated with the trait of weaning weight, and all the loci were significant at the chromosome level. Among them, one exhibited significance at the genome-wide level (given 0.5/718040 = 6.96E-07, i.e., −log10(0.5/718040) = 6.16), which was located on chromosome 27. Among the other 20 loci, 10 were located on chromosome 27, 2 were located on chromosome 11, and the others were located on chromosomes 10, 14, 15, 16, 18, 19, 20, and 26 (Supplementary Table S6). Figure 2d. illustrates the Manhattan plots of the GWAS for the trait of weaning weight.

Twenty-one loci were significantly correlated with the daily weight gain before weaning, and all the loci were significant at the chromosome level. Among them, 4 were located on chromosomes 11 and 27, while 2 were located on chromosomes 1, 19, and 25; the other loci were located on chromosomes 6, 10, 12, 20, 23, and 24. Additionally, one locus was located in an unknown chromosome (Supplementary Table S7). Figure 2e presents the Manhattan plots of the GWAS for the trait of daily weight gain before weaning.

The structure of a group, that is, the hierarchy of a group, indicates that subgroups with varying gene frequencies exist in the studied group. Samples within one subgroup have closer relatedness, whereas those from different subgroups have more distant relatedness. Hierarchical analysis of a group can quantify the number of ancestors in the studied group and infer the consanguinity origin of each sample. Currently, it is used as an analytical clustering method for groups with more applications, which contributes to the understanding of the evolution of samples.

**Figure 3:**
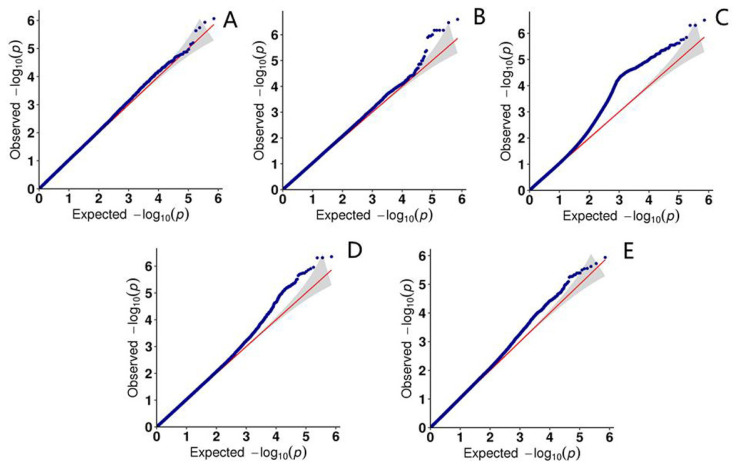
Quantile-quantile plot of the different traits. The abscissa represents the expected value, and the ordinate represents the observed value. The red line in the figure represents the 45° center line, and the gray area is the 95% confidence interval of the scattered points on the graph.

Using R language, the quantile-quantile plot was constructed for five traits (singleton or twins, birth weight, age [in days] at sexual maturity, weaning weight, and daily weight gain from birth to weaning). This plot can give an intuitionistic reflection of the differences between the observed and predicted values (Figure 3).

### Analysis of linkage disequilibrium

**Figure 4:**
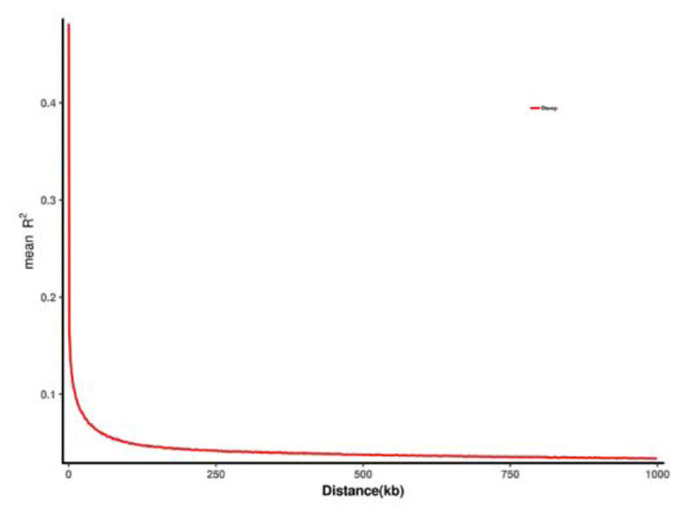
Linkage disequilibrium decay attenuation diagram.

For one chromosome, the LDs for each pair of SNPs were calculated within the range of a certain distance (e.g., 1,000 kb). The intensity of LD was expressed as an r^2^ value, and an r^2^ value closer to 1 indicated a more intense disequilibrium of linkage. Spanning distance along with the maximum r^2^’ decreasing to its half value was defined as LD decay distance. Plink2 [7] software was used for LD analysis. A decay diagram of LD is depicted in Figure 4.

### Data digging for candidate genes

**Figure 5:**
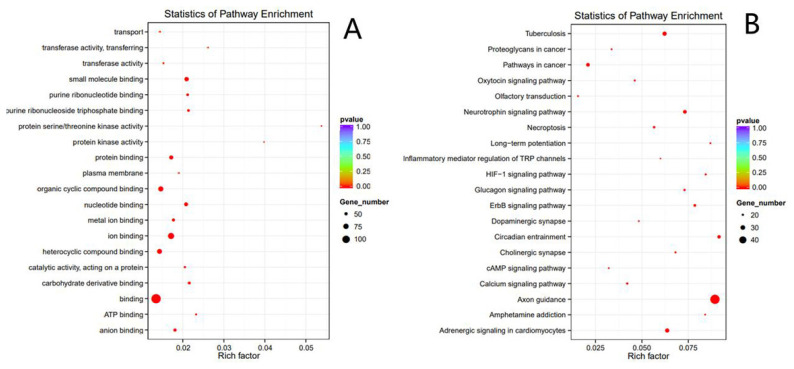
Cluster analysis of candidate genes. The q value on the right represents the p-value; the lower the p-value, the redder the color, and the more significant the enrichment; the size of the circles represents the number of genes; the larger the gene represents the pathway or gene ontology (GO) term, the more the genes contained in each circle. On the left is a detailed description of the pathway or GO term. (A) The GO enrichment map; (B) the Kyoto encyclopedia of genes and genomes enrichment map.

The selected 151 SNPs were assessed through GO and KEGG analyses for function annotation and enrichment analysis on genes using GSEA analytical tools and clusterProfiler software. The interaction network diagram for candidate genes was then plotted based on known interactions of the sheep genome (Ovis_aries_v1.0). Enrichment analysis is based on the hypergeometric distribution theory, and the obtained set of differentially expressed genes is the set of differentially expressed genes after difference significance analysis and with annotations in the GO or KEGG database. Background genes’ set refers to the set of all genes subjected to difference significance analysis and annotated in the GO or KEGG database (Figure 5). As shown in Figure 5, through function annotation and function clustering analyses for candidate genes, the functions of candidate genes were accumulated mainly in nucleotide metabolism, metal ion complexation, circadian rhythms, oxytocin signaling pathway, and neurotrophin signaling pathway.

**Figure 6:**
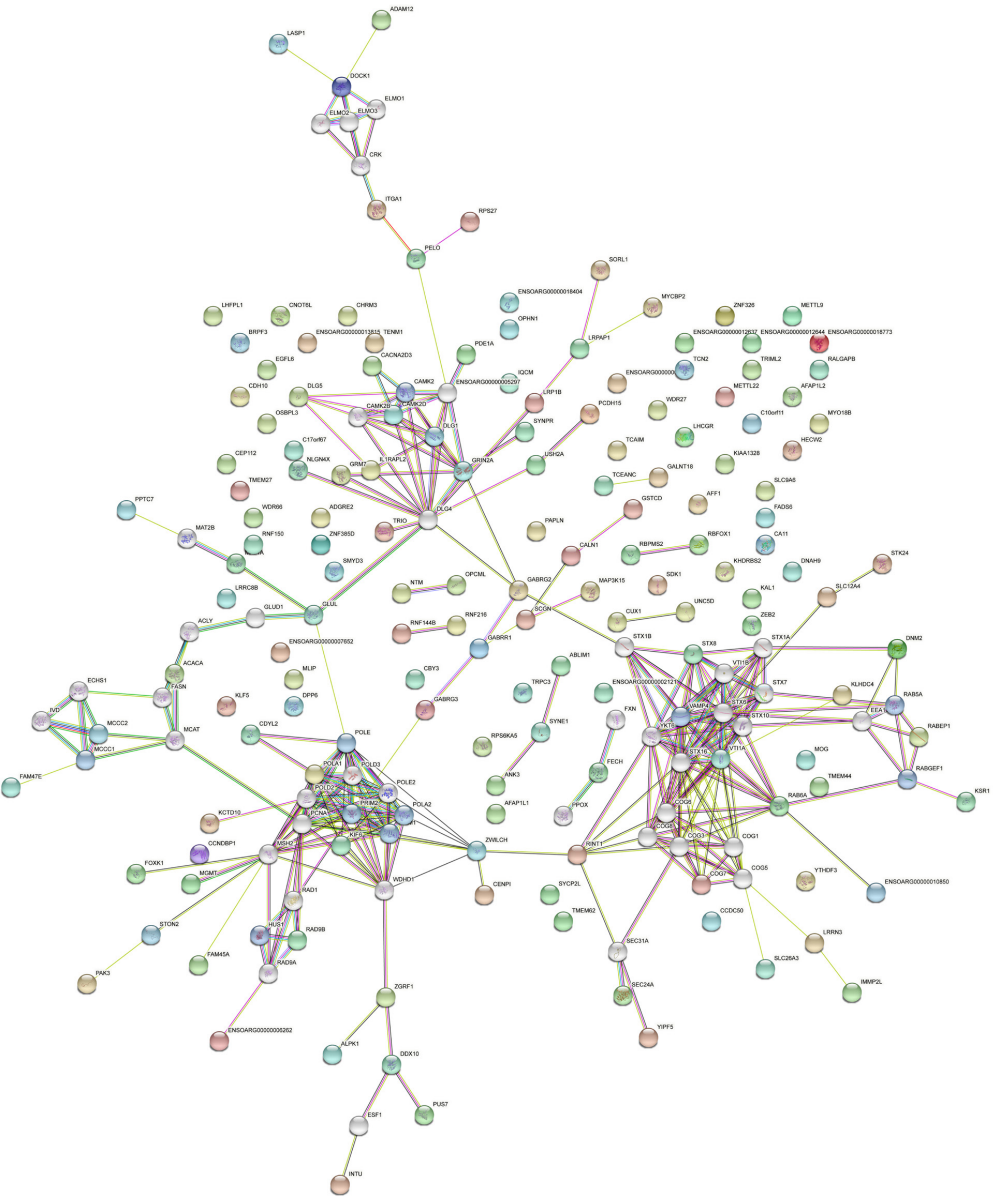
Interaction network diagram of candidate genes.

Interaction network analysis was performed for genes and proteins mainly through the protein-protein interactions in the STRING database (https://string-db.org/). Overall, 395 genes were associated with the five traits, and their corresponding protein sequences were extracted to screen optimal genes for constructing a diagram of genes’ interactions (Figure 6).

## DISCUSSION

The GWAS is performed to screen high-density molecular markers (most of which are SNPs) at the genome-wide level, use statistical methods to perform association analysis between the obtained information about molecular markers and phenotypic traits, combine the results with the genetics theory to screen and validate genetic variations related to targeted traits, and then select the candidate genes relevant to targeted traits. Compared with the traditional candidate gene method, the GWAS method can locate the mutation regions more accurately and has become a research hotspot in the field of genetics and breeding at home and abroad. Therefore, GWAS has become an effective method for identifying candidate genes relevant to the complex traits of animals and plants as well as human diseases. Among the various traits of livestock, the reproductive trait has significant economic benefits but involves extremely complex genetic mechanisms and greater heritability, and the phenotype of the reproductive trait could be revealed only after birth. Hence, genetic advances could be achieved very slowly through traditional breeding methods. Even for livestock such as pigs with great reproductive ability, slower improvements have been observed in their reproductive traits. By contrast, the GWAS method enables the QTL regions and candidate genes to be localized more accurately. It can also select genetic markers relevant to breeding and reproduction more precisely and enable its direct application in breeding, thus accelerating the breeding process. As for singleton animals of cows, sheep, and goats, the GWAS method could be used to select and localize candidate genes or genome regions associated with reproductive traits. This is more remarkable for applying genomic selection breeding techniques and enhancing the reproduction efficiency of the corresponding species.

In the GWAS of 200 ewes of Qianhua mutton merino, 151 SNP loci exhibiting significance at the chromosome level were identified. Among them, 3 showed significance at the genome-wide level, 2 of which were located on chromosomes 8 and 14. Additionally, the annotation results revealed that two loci were located on the *SYNE1* and *SLC12A4* genes, which are genes associated with birth weight. One locus was located on chromosome 27, and the annotation results revealed that it was located on LOC101121916 (related to weaning weight). Based on these analytical results, we can preliminarily deduce that these genes are vital candidate genes influencing the reproductive traits of Qianhua mutton merino.

Spectrin repeat-containing nuclear envelope protein (*SYNE*) 1, also called the *Nesprin-1* gene, encodes a spectrin repeat sequence that encodes proteins expressed in the skeletal muscle, smooth muscle, and peripheral blood lymphocytes. The protein is expressed and localized in the karyotheca, Golgi apparatus, and cytoskeleton, including the central nervous system [8]. This protein is characterized by multiple repeat sequences of various spectrins highly expressed in the striated muscle [9]. It participates in the processes of cell proliferation and division (including cytokinesis and localization of the nucleus), and the related pathways include meiosis, cell cycle, and mitosis. GO annotations related to this gene are involved in binding with nucleotides, and *SYNE2* is an important paralogue of this gene. The most prominent characteristic of SYNE1 is its size, and it encodes a protein comprising approximately 8,797 amino acid residues (41,000 kDa). The encoded protein contains two actin-binding domains at the N-terminal, which consist of a tandem pair of calmodulin homologous domains, a transmembrane domain, several spectrin repeat sequences, and a C-terminal Klarsicht structural domain (KASH). Syne-1 is involved in the anchoring of the special muscle nucleus at the neuromuscular junction (NMJ) [10]. Higher vertebrates may have additional nuclear migration compensation mechanisms. Noteworthy, the muscle nucleus of muscle tissues from affected individuals exhibited abnormal localization at the NMJ. However, clinically or electro-physiologically detectable muscle phenotypes were not detected in the affected individuals. Abnormal aggregation of the postsynaptic nucleus detected in affected individuals presented no critical effects on the maturity or maintenance of the NMJs. All members of the spectrin family, including spectrin, dystrophin, and utrophin, seem to share a common function: they enable linkages between the plasmalemma and actin cytoskeleton. The GWAS findings in this study showed that SYNE1 was significantly correlated (at the genome-wide level) with the trait of birth weight in Qianhua mutton merino. Nevertheless, the specific functioning mechanisms remain to be confirmed in subsequent studies.

Solute carrier family 12 (*SLC12*) member 4, also called potassium/chloride transporter protein, is an important ion transporter protein–encoding gene. It also encodes proteins that mediate the coupled movement of potassium and chloride ions across the plasmalemma. Members of the SLC family have shown modulatory effects on exocytosis. Nonetheless, the functions of the SLC12 family have not been ascertained [11]. Hanzawa et al [12] reported the localization of SLC12A4 and SLC7A10 homologs in horses, features of molecular polymorphisms, and relationships between these genes as well as *SLC7A9* and variability of erythrocyte osmotic fragility in horses. However, only few studies have focused on SLC12A4 in sheep.

Bone morphogenetic protein 2 inducible kinase (*BMP2K*), that is, BMP2-induced kinase, is protein-encoding gene. Bone morphogenetic proteins (BMPs) are a type of secretory protein participating in the regulation of organisms’ functions such as cell proliferation and differentiation, apoptosis, morphogenesis, and organofaction. Among these functions, BMP2 is closely related to bone growth and differentiation [13]. The *BMP2* gene also promotes the differentiation of preadipocytes in sheep, thereby participating in fat deposition in sheep tail and further influencing the development of sheep tail shape [14]. In addition to cell cycle modulation, BMP2K may participate in the differentiation of megakaryocytes through other modulating mechanisms.

Inner mitochondrial membrane peptidase subunit 2 (IMMP2L) is a type of mitochondrial protein located in the mitochondria. This protein is essential for the catalytic activity of the mitochondrial intimal peptidase (IMP) complexes. It can cleave the signal peptide sequence of mitochondrial cytochrome C1 (cyc1) and glycerophosphate dehydrogenase 2 (GDP2). Mutations in IMMP2L can increase the risk of cerebral injury induced by transient cerebral ischemia, inhibit and decrease cellular activity, promote ROS generation, and reduce mitochondrial membrane potential [15]. Thus, it could lead to a decrease in food intake and weight loss in animals [16]. KEGG annotations related to this gene include ovarian follicle development, female gamete generation, ovulation, and so on.

Zinc finger protein 326 (*ZNF326*), a member of the protein family AKAP95, is a gene encoding zinc finger transcription factor proteins. It is a new partner for interactions and substrate of the PRMT5/WDR77 nuclear complexes. ZNF326 interacts with DBC1 and promotes the expression of matrix metalloproteins (MMPs), cyclins, and factors associated with epithelial-mesenchymal transition (EMT) [17].

Sortilin-related receptor 1 (SORL1), also named LR11 or SORLA, is a membrane-bound protein. The encodes heterozygous receptors with several domains involved in endosomal intracellular sorting and transport of the proteins to the corresponding subcellular compartments, mainly located in endosomes and Golgi apparatus [18]. The encoded proteins include repeat sequences of fibronectin (type III) and epidermal growth factors. The proprotein is converted to mature receptors after protein hydrolysis, and these mature receptors may play a role in endocytosis and sorting. It is mainly located in the lysosomal system of cells, trans-Golgi network, and mitochondria-associated membrane [19] of the middle endoplasmic reticulum. It is also expressed in other tissues, such as testis, ovary, thyroid, and lymph nodes [20].

Acetyl-CoA carboxylase alpha (ACACA) mainly converts acetyl-CoA into malonyl-CoA through carboxylation and acts as a rate-limiting enzyme for the synthesis of fatty acids. Therefore, it is usually used as a marker to evaluate the differentiation degree of adipocytes of intramuscular fat (IMF) in animals and candidate genes [21]. ACACA participates in the development of fat tissues and the metabolism of lipids [22]. Based on this theory, it has been used to study whether it could be used as molecular markers for traits of pork quality [23].

LASP1 (LIM and SH3 protein 1) is a scaffolding protein that mediates cell migration, proliferation, and survival in several human breast cancer cell lines [24]. This gene encodes members in the protein subfamily of LIM characterized by the sequence motif of LIM and the structural domain of Src homologous region 3. It is also a member of the actin-binding protein family, and the encoded proteins are dependent on the signaling proteins of cAMP and cGMP. The elongated regions of the encoded proteins are combined with the actin cytoskeleton of the cell membrane. In human macrophages, LASP1 is associated with cyclic-F-actin in corpuscles, which promotes protein hydrolysis in the extracellular matrix [25]. At present, studies on LASP1 have focused mostly on human cancers, and the underlying mechanisms remain unclear.

Secretagogin (SCGN) is a calcium-binding protein found in the cytoplasm, and this protein is involved in KClstimulated calcium flux, cell proliferation, and insulin release from the pancreas. It is expressed in the interneurons of the glomerulus and granular cell layers, thyroid, gastrointestinal tract, adrenal medulla, paranephros, and brain [26]. The exact mechanisms for the effects of CGN on insulin release have not been known until recent years. It involves SGN-interacting proteins, which are actin-binding proteins participating in the transport and exocytosis of insulin granules [27] or modulating actin cytoskeleton by promoting the transport of vesicles toward the periphery during insulin release [28].

A disintegrin and metalloprotease 12 (ADAM12) is a member of the multi-domain metalloproteinases-bisintegrin family. It has characteristics of cell binding and metalloproteinases and participates in several biological processes related to the cell-cell and cell-extracellular matrix interactions (including fertilization, muscle development, and neurogenesis). Diseases associated with ADAM12 include lung cancer and ectopic gestation, and the related pathways include RET signal transduction and G-protein–coupled receptor signal transduction. During the early stage of mammalian development, ADAM12 mRNA expression is significant in mesenchymal cells, while mesenchymal cells would develop into skeletal muscle, bones, and visceral organs [29]. In adult mammals, ADAM12 has shown high expression in tissues, and its expression is the highest in bones [30].

In addition to analyses for annotated candidate genes of the aforementioned significant SNP loci, we performed gene function annotation and clustering analyses for the other 139 SNP loci that exhibited significance at the chromosome level. In the past two decades, research on the application of the GWAS method for the association analysis of growth, development, and reproduction traits of sheep has gained wide focus for selecting and localizing corresponding candidate genes. Zhang et al [31] used 50KSNP chips of sheep to conduct GWAS on Sunit sheep, German merino, and Dorper sheep and concluded that 36 SNPs were significantly correlated with seven traits (birth weight, weight at the age of 6 months, daily weight gain before weaning, daily weight gain, chest circumference, and tube circumference). Among these SNPs, 10 were significantly correlated (at the genome-wide level) with daily weight gain before weaning; based on gene annotations, five candidate genes associated with daily weight gain before weaning were *MEF2B*, *RFXANK*, *CAMKMT*, *TRHDE*, and *RIPK2*. Gholizadeh et al [32] conducted GWAS for birth weight, weaning weight, weight at the age of 6 months, and weight upon the first birthday in Baluchi sheep. Concomitantly, the authors found that 13 SNPs were significant at the chromosome level; gene annotations indicated that the *STRBP* and *TRAMIL* genes were candidate genes for birth weight, while *APIP* and *DAAMI* were candidate genes for weight at the age of 3 months. By contrast, Al-Mamun et al [33] found that 39 SNPs were associated with body weight in Australian merino; among these SNPs, 13 related to body weight were located in the zone of 36.15 to 38.56 Mb on chromosome 6, and this zone involved candidate genes for body weight (including *LAP3*, *NCAPG*, and *LCORL*). Demars et al [34] used the GWAS method and analyzed the lambing traits of French Grivette sheep and Polish Olkuska sheep and confirmed that *BMP15* was an important candidate gene determining the number of lambs birthed. Based on 50KSNP chips of sheep, Våge et al [35] applied GWAS for the lambing numbers of female generations of Norwegian white sheep rams and found that the best candidate gene that could effectively increase the ovulation rate or lambing birthing was located in chromosome 5 of sheep (near the *GDF9* gene). Gholizadeh et al [36] applied GWAS for 96 individuals from two groups of Baluchi sheep according to the rate of twin production in the first four generations and concluded that SNPs influencing the total number of produced lambs were located on chromosomes 10 and 15.

In this study, GWAS was used, and the results revealed that 151 SNPs in Qianhua mutton merino were correlated (at the chromosome level) with five traits (i.e., traits of singleton or twins, birth weight, age [in days] at sexual maturity, weaning weight, and daily weight gain from birth to weaning) of its ewe groups. Among the SNPs, two were located on chromosomes 8 and 14 (associated with birth weight) and 1 was located on chromosome 27 (associated with weaning weight), exhibiting significance at the genome-wide level. All the other 148 SNPs presented significant correlations (at the chromosome level) with the five traits, which were located on the other chromosomes. These results were similar to those of other relevant traits of sheep [37], pig [38], and cow [39], suggesting that GWAS was efficient in exploring significant loci of the targeted traits at the genome-wide level, which cannot be replaced by traditional methods.

In this study, we performed GO and KEGG functional annotation and enrichment analysis for 151 SNPs of five reproduction-related traits of Qianhua mutton merino. Genes associated with these traits were *SYNE1*, *SLC12A4*, *BMP2K*, *CAMK2D*, *MMP2L*, *ZNF326*, *PELO*, *SORL1*, *ACACA*, *LASP1*, *SCGN*, *ADAM12*, and other genes near the 151 SNP loci related to reproduction traits. Among these genes, ACACA mainly converts acetyl-CoA into malonyl-CoA by carboxylation, functioning as a rate-limiting enzyme during the synthesis of fatty acids. Therefore, this gene is usually used as a marker for evaluating the differentiating degree of adipocytes of IMF in animals and candidate genes [21]. It also participates in the development of fat tissues and lipid metabolism [22]. ZNF326 could interact with DBC1, thus promoting the expression of MMPs, cyclins, and factors associated with EMT [17]. Proteins encoded by IMMP2L are located in the mitochondria. IMMP2L is essential for the catalytic activity of IMP complexes. IMMP2L is involved in ovarian follicle development, generation of the female gamete, ovulation, and so on. CAMK2D belongs to the family of serine/threonine protein kinases and the subfamily of calmodulin-dependent protein kinase. GO annotations for this gene include serine/threonine protein kinase activity, purine ribonucleotide binding, homologous dimerization of proteins, and protein kinase activity. Relevant KEGG annotations include ErbB signaling pathway, HIF-1 signaling pathway, meiosis of oocytes, and oxytocin signaling pathway. The encoded protein contains two actin-binding domains at the N-terminal, which consist of a tandem pair of calmodulin homologous domains, a transmembrane domain, several spectrin repeat sequences, and a C-terminal KASH. This protein is characterized by highly expressed repeat sequences of various spectrins in the striated muscle [9] and participates in cell proliferation and division (including cytokinesis and localization of nucleus), and the related pathways include meiosis, cell cycle, and mitosis. Moreover, it is involved in the anchoring of the special muscle nucleus at the NMJ and provides linkages between the plasmalemma and actin cytoskeleton [10].

The SNP loci selected in this study are mainly enriched in nucleotide metabolism, metal ion binding, circadian rhythm, oxytocin signaling pathway, and neurotrophin signaling pathway. Therefore, genes associated with these signaling pathways are presumed to be candidate genes correlated with reproduction-related traits in sheep. However, reproduction-related traits in sheep are complex quantitative traits controlled by various genes. As such, it remains unclear whether candidate genes selected in this study could directly affect the targeted traits, which needs to be further confirmed.

## CONCLUSION

Using GWAS, 151 SNP loci were detected for five traits related to reproduction (including singleton or twins, birth weight, age [in days] at sexual maturity, weaning weight, and daily weight gain from birth to weaning). Among the SNP loci, 3 exhibited significance at the genome-wide level, which were correlated with birth weight and weaning weight. GO and KEGG functional annotation and enrichment analyses were conducted for candidate genes. The results showed that functions of these candidate genes were mainly enriched in nucleotide metabolism, metal ion binding, circadian rhythm, oxytocin signaling pathway, and neurotrophin signaling pathway. Overall, 151 candidate genes of *SYNE1*, *SLC12A4*, *BMP2K*, *CAMK2D*, *IMMP2L*, *DMD*, and *BCL2* were obtained through comparative genomics on the basis of reference to the sheep genome Ovis_aries_v1.0 information.

## Data Availability

The original contributions presented in the study are included in the article/supplementary material, further inquiries can be directed to the corresponding author/s. The datasets have been submitted to the SRA database of NCBI (accession number PRJNA1065116).

## SUPPLEMENTARY MATERIAL

**Supplementary Table S1**. Data of the reproductive traits Qianhua Mutton Merino sheep

**Supplementary Table S2**. Bonferroni method to calculate the significant threshold at the chromosome and genome-wide levels

**Supplementary Table S3**. Results of the genome-wide association analysis of singleton and twin traits of Qianhua mutton merino

**Supplementary Table S4**. Results of the genome-wide association analysis of birth weight in Qianhua mutton merino

**Supplementary Table S5**. Results of the genome-wide association analysis of age at sexual maturity in Qianhua mutton merino

**Supplementary Table S6**. Results of the genome-wide association analysis of weaning weight in Qianhua mutton merino

**Supplementary Table S7**. Results of the genome-wide association analysis of daily weight gain before weaning in Qianhua mutton merino
