# Supplementary material for: Genome-wide association research on the reproductive traits of Qianhua Mutton Merino sheep
Source: Anim Biosci. 2024 Apr 1;37(9):1535–47. doi: 10.5713/ab.23.0365 (PMC11366534; doi:10.5713/ab.23.0365)
Supplement: Supplementary file 1 [file ab-23-0365-Supplementary-Table-1.pdf]

Supplementary Tables

Table S1. Data of the reproductive traits Qianhua Mutton Merino sheep

No.	singleton(1) / twins(2)	birth weight (kg)	weaning weight (kg)	age [in days] at sexual maturity	daily weight gain from birth to weaning (g/d)
1	1	4.6	22.1	613	194.44
2	1	4.4	29.5	578	278.89
3	2	3.2	21.7	597	205.56
4	1	5	20.8	560	175.56
5	1	4.9	23.6	584	207.78
6	1	5.5	22.8	626	192.22
7	1	6.2	21.3	601	167.78
8	2	4.2	22.6	583	204.44
9	1	4.5	22.3	623	197.78
10	1	4.5	26.2	594	241.11
11	1	5.2	21.3	627	178.89
12	1	5.3	22.6	575	192.22
13	1	4.6	22.3	516	196.67
14	1	4.6	22	619	193.33
15	1	3.6	21.4	556	197.78
16	1	4.5	21.6	601	190.00
17	1	3.9	22.9	574	211.11
18	1	4.4	21.5	587	190.00
19	1	5.2	19.6	633	160.00
20	2	3.7	19.4	592	174.44
21	1	4.2	21.6	581	193.33
22	1	4.2	25.8	595	240.00
23	2	4	23.4	605	215.56
24	1	3.9	20.4	592	183.33
25	1	4	23.8	580	220.00
26	1	3.9	25.8	599	243.33
27	1	3.8	21.4	624	195.56
28	1	4.7	28.8	585	267.78
29	2	3.8	23.4	607	217.78
30	1	4.3	22.6	630	203.33
31	1	5	21.4	582	182.22
32	2	3.9	25.6	594	241.11
33	1	4.8	26	615	235.56
34	1	4.9	25.6	597	230.00
35	1	4.3	20.2	606	176.67
36	2	4.5	21	582	183.33
37	1	5.3	19.2	632	154.44
38	1	5	17.6	593	140.00

39	1	5	25.2	569	224.44
40	2	3.8	19.6	631	175.56
41	1	3.4	22	584	206.67
42	2	4.2	21.5	604	192.22
43	1	5	20.4	632	171.11
44	1	4.2	27.6	598	260.00
45	1	5	21.8	577	186.67
46	1	4.1	25	602	232.22
47	2	3.5	22	625	205.56
48	1	4.3	21.6	591	192.22
49	1	4.2	21.4	582	191.11
50	1	4.2	23.6	627	215.56
51	1	4.2	18.4	592	157.78
52	1	4.3	23.6	620	214.44
53	2	4.2	20.2	626	177.78
54	2	3.5	19.2	634	174.44
55	2	4.2	18.6	583	160.00
56	1	4.1	24.8	601	230.00
57	1	4.6	19.4	618	164.44
58	1	3.9	25.4	599	238.89
59	2	4.2	17.4	572	146.67
60	2	3.4	25.4	630	244.44
61	1	4.2	22.5	558	203.33
62	1	4.2	21.4	629	191.11
63	2	4.1	21.4	563	192.22
64	1	4.6	22.3	629	196.67
65	1	4.2	22.8	573	206.67
66	1	4.4	21.7	625	192.22
67	2	3.2	22.4	598	213.33
68	2	3.9	17.8	594	154.44
69	1	3.8	24.6	588	231.11
70	2	4.2	23.2	642	211.11
71	1	4.2	31.2	608	300.00
72	1	4.8	17.2	599	137.78
73	1	4.2	22.6	616	204.44
74	1	4.5	18.2	589	152.22
75	1	4.8	22.8	626	200.00
76	1	4.4	21.6	621	191.11
77	2	3.9	23.4	583	216.67
78	1	4.6	20.6	594	177.78
79	2	4	21	609	188.89
80	1	4.2	19.2	588	166.67
81	2	4.2	19.6	617	171.11
82	1	3.8	22.1	599	203.33

83	1	4.4	22.9	629	205.56
84	1	4.4	21.6	630	191.11
85	1	4.2	23.3	593	212.22
86	1	3.9	25.6	627	241.11
87	1	5.2	24.4	631	213.33
88	1	4.1	22.5	601	204.44
89	1	4.4	19.8	579	171.11
90	1	4.6	21.9	568	192.22
91	1	4.1	22.4	597	203.33
92	1	4.1	23.6	624	216.67
93	1	3.8	20.7	588	187.78
94	2	4.2	17.6	580	148.89
95	1	4.2	17.2	619	144.44
96	1	4.5	23.6	598	212.22
97	1	4.9	23.2	596	203.33
98	1	4.1	21.2	604	190.00
99	1	4.2	20.6	615	182.22
100	1	4.3	22.1	568	197.78
101	1	4.6	21.8	620	191.11
102	2	4.1	22.7	626	206.67
103	2	4.4	19.8	603	171.11
104	1	4.8	21.9	588	190.00
105	2	4	20.6	591	184.44
106	1	4.3	25.2	570	232.22
107	1	5	23.8	503	208.89
108	1	5.3	25.6	592	225.56
109	2	4	20.4	600	182.22
110	1	4.7	21	641	181.11
111	1	5.2	20	596	164.44
112	1	4.3	23.4	612	212.22
113	1	5.1	17	604	132.22
114	1	5.1	27	593	243.33
115	1	4.3	28.6	647	270.00
116	1	5	28.2	602	257.78
117	1	5	22.4	597	193.33
118	1	5.1	18.6	634	150.00
119	1	5.3	27.2	619	243.33
120	1	5.2	23.6	574	204.44
121	2	4.2	25.2	604	233.33
122	1	4.7	25.4	627	230.00
123	1	4.6	23.6	594	211.11
124	2	4.2	21.4	611	191.11
125	1	4.6	22.1	594	194.44
126	1	4.4	21.4	592	188.89

127	1	5	24.2	607	213.33
128	2	4.5	22.3	588	197.78
129	1	5.2	23.6	601	204.44
130	2	4.5	22.8	632	203.33
131	1	4.9	21.6	621	185.56
132	1	4.7	22.4	575	196.67
133	1	5.4	21	594	173.33
134	1	5.1	21	594	176.67
135	1	5.3	20.2	605	165.56
136	1	5	20	610	166.67
137	2	4.4	20	579	173.33
138	1	5.3	24.4	641	212.22
139	1	5.1	24	596	210.00
140	2	4	21.6	604	195.56
141	1	5.1	23.6	562	205.56
142	1	5	22	583	188.89
143	1	5.1	27.4	597	247.78
144	1	4.3	22.6	576	203.33
145	1	5	21.2	631	180.00
146	1	5.3	21.2	592	176.67
147	1	5.1	21.2	603	178.89
148	2	4.3	25.2	586	232.22
149	1	4.3	23.6	633	214.44
150	2	4.2	20.2	597	177.78
151	1	5	23.4	576	204.44
152	1	5	22	642	188.89
153	1	4.8	21.4	622	184.44
154	1	4.2	22	607	197.78
155	1	4.5	22.3	588	197.78
156	1	5.1	25.4	591	225.56
157	1	5	24.6	615	217.78
158	2	4.2	20.6	572	182.22
159	1	5	21.4	595	182.22
160	1	5.3	22	576	185.56
161	2	4.3	22.8	603	205.56
162	2	4.1	21.8	596	196.67
163	1	4.5	21.2	611	185.56
164	2	4.4	19.8	603	171.11
165	2	4.2	28	594	264.44
166	2	4.3	23.4	551	212.22
167	1	4.2	18.6	597	160.00
168	1	4.8	23	592	202.22
169	2	4.1	24.4	636	225.56
170	1	4.5	22.3	558	197.78

171	1	4.6	24	595	215.56
172	2	4.3	23.2	610	210.00
173	1	5	24.8	602	220.00
174	1	5	20.6	639	173.33
175	1	5.1	21.4	587	181.11
176	1	4.6	23.2	591	206.67
177	1	5.4	21.2	573	175.56
178	2	4	21	615	188.89
179	2	4.4	19.6	604	168.89
180	1	4.7	23.8	597	212.22
181	1	5	19.4	605	160.00
182	2	4.4	21.4	570	188.89
183	2	4.4	20.6	582	180.00
184	2	4.1	18.2	637	156.67
185	2	4.5	23.4	603	210.00
186	2	4.4	20.8	589	182.22
187	1	4.9	21.9	612	188.89
188	1	5	22.1	606	190.00
189	2	4.2	23.8	614	217.78
190	1	4.4	22	595	195.56
191	2	4.7	20.4	638	174.44
192	2	4	19	571	166.67
193	1	5.3	24.6	598	214.44
194	1	5.2	20.6	587	171.11
195	2	3.5	17.2	617	152.22
196	2	3.6	18.6	588	166.67
197	2	4.5	21.2	600	185.56
198	2	4.5	15.8	624	125.56
199	2	3.5	18.8	564	170.00
200	2	4.4	17	595	140.00
