# Supplementary material for: Genome-wide association research on the reproductive traits of Qianhua Mutton Merino sheep
Source: Anim Biosci. 2024 Apr 1;37(9):1535–47. doi: 10.5713/ab.23.0365 (PMC11366534; doi:10.5713/ab.23.0365)
Supplement: Supplementary file 2 [file ab-23-0365-Supplementary-Table-2.pdf]

Table S2. Bonferroni method to calculate the significant threshold at the chromosome and genome-wide levels.

chromosome	Chromosome ID	SNP number	P Value
1	NC_040252.1	75,026	6.66436E-06
2	NC_040253.1	66,994	7.46335E-06
3	NC_040254.1	60,139	8.31407E-06
4	NC_040255.1	33,616	1.48739E-05
5	NC_040256.1	29,441	1.69831E-05
6	NC_040257.1	34,225	1.46092E-05
7	NC_040258.1	27,057	1.84795E-05
8	NC_040259.1	24,062	2.07797E-05
9	NC_040260.1	27,505	1.81785E-05
10	NC_040261.1	24,206	2.0656E-05
11	NC_040262.1	16,033	3.11857E-05
12	NC_040263.1	22,105	2.26193E-05
13	NC_040264.1	23,459	2.13138E-05
14	NC_040265.1	18,692	2.67494E-05
15	NC_040266.1	23,204	2.1548E-05
16	NC_040267.1	20,529	2.43558E-05
17	NC_040268.1	21,915	2.28154E-05
18	NC_040269.1	18,254	2.73913E-05
19	NC_040270.1	17,337	2.88401E-05
20	NC_040271.1	15,700	3.18471E-05
21	NC_040272.1	15,284	3.27139E-05
22	NC_040273.1	15,535	3.21854E-05
23	NC_040274.1	19,238	2.59902E-05
24	NC_040275.1	13,089	3.82E-05
25	NC_040276.1	14,328	3.48967E-05
26	NC_040277.1	14,494	3.4497E-05
27	NC_040278.1	23,289	2.14694E-05
0	-	3284	0.000152253
Total	-	718040	6.9634E-07

Note: “0” represents the significance threshold at the chromosome level where an SNP with the unknown position is located. “Total” represents the significant threshold at the genome-wide level.
