# Supplementary material for: Genome-wide association research on the reproductive traits of Qianhua Mutton Merino sheep
Source: Anim Biosci. 2024 Apr 1;37(9):1535–47. doi: 10.5713/ab.23.0365 (PMC11366534; doi:10.5713/ab.23.0365)
Supplement: Supplementary file 3 [file ab-23-0365-Supplementary-Table-3.pdf]

Table S3. Results of the genome-wide association analysis of singleton and twin traits of Qianhua mutton merino.

No.	Chr.	Chr.ID	Pos	Start(bp)	End(bp)	Position(bp)	P-value	Genes
1	6	NC_040257.1	103548932	103540594	103662068	121474	8.60E-07	BMP2K
2	2	NC_040253.1	137498732	137586848	137879528	292680	1.17E-06	PDE1A
3	12	NC_040263.1	21148483	20870805	21816602	945797	4.81E-06	USH2A
4	5	NC_040256.1	1589256	1469382	1529399	60017	6.28E-06	CBY3
5	25	NC_040276.1	32280293	32271931	33415964	1144033	6.64E-06	LRMDA
6	6	NC_040257.1	112262122	112165132	112364250	199118	9.67E-06	AFF1
7	6	NC_040257.1	14778768	14727688	15282831	555143	1.07E-05	CAMK2D
8	6	NC_040257.1	16190170	16167634	16223214	55580	1.31E-05	ZGRF1
9	26	NC_040277.1	28996591	29093810	29171413	77603	1.59E-05	RBPMS
10	24	NC_040275.1	9035775	8747719	9187886	440167	1.84E-05	GRIN2A
11	11	NC_040262.1	33013083	32830528	33115909	285381	1.93E-05	DNAH9
12	20	NC_040271.1	14820100	14747546	14754042	6496	2.53E-05	KIF6
13	21	NC_040272.1	11740039	10897315	12643369	1746054	2.66E-05	LOC114110160
14	21	NC_040272.1	35859388	35632919	36586857	953938	3.08E-05	NTM
