# Supplementary material for: Genome-wide association research on the reproductive traits of Qianhua Mutton Merino sheep
Source: Anim Biosci. 2024 Apr 1;37(9):1535–47. doi: 10.5713/ab.23.0365 (PMC11366534; doi:10.5713/ab.23.0365)
Supplement: Supplementary file 4 [file ab-23-0365-Supplementary-Table-4.pdf]

Table S4. Results of the genome-wide association analysis of birth weight in Qianhua mutton merino.

No.	Chr.	Chr.ID	Pos	start(bp)	End(bp)	Position(bp)	P-value	Genes
1	8	NC_040259.1	83500583	83232480	83724150	491670	2.53E-07	SYNE1
2	14	NC_040265.1	38206765	38196268	38199357	3089	3.38E-07	SLC12A4
3	2	NC_040253.1	212425891	212197473	212454923	257450	2.03E-06	HECW2
4	5	NC_040256.1	13571112	13616993	13632918	15925	2.45E-06	DNM2
5	2	NC_040253.1	177018183	177117351	177561106	443755	3.85E-06	ZEB2
6	1	NC_040252.1	214470276	214414602	214497248	82646	4.43E-06	CCDC50
7	7	NC_040258.1	106945458	106912592	107007246	94654	4.52E-06	RPS6KA5
8	20	NC_040271.1	11801153	11643679	11713858	70179	4.59E-06	BRPF3
9	23	NC_040274.1	23885583	23523398	23841349	317951	6.64E-06	KIAA1328
10	7	NC_040258.1	88476708	88457379	88489145	31766	8.42E-06	PAPLN
11	4	NC_040255.1	52632339	52634533	52658079	23546	8.48E-06	RINT1
12	20	NC_040271.1	798277	200567	1474218	1273651	1.10E-05	KHDRBS2
13	9	NC_040260.1	44502267	44397191	44428710	31519	1.15E-05	YTHDF3
14	27	NC_040278.1	141876976	141856904	141857740	836	1.22E-05	CENPI
15	6	NC_040257.1	16362516	16277142	16387536	110394	1.42E-05	ALPK1
16	16	NC_040267.1	64196012	64030354	64344502	314148	1.67E-05	TRIO
17	16	NC_040267.1	52691034	52635014	52821486	186472	2.00E-05	LOC101117364
18	11	NC_040262.1	37202989	37247418	37269753	22335	2.08E-05	RABEP1
19	17	NC_040268.1	41811857	41761438	41835223	73785	2.12E-05	TRPC3
20	25	NC_040276.1	36781373	36726518	36737185	10667	2.58E-05	MAT1A
21	24	NC_040275.1	5973633	5478796	6845963	1367167	2.92E-05	RBFOX1
