# Supplementary material for: Genome-wide association research on the reproductive traits of Qianhua Mutton Merino sheep
Source: Anim Biosci. 2024 Apr 1;37(9):1535–47. doi: 10.5713/ab.23.0365 (PMC11366534; doi:10.5713/ab.23.0365)
Supplement: Supplementary file 5 [file ab-23-0365-Supplementary-Table-5.pdf]

Table S5. Results of the genome-wide association analysis of age at sexual maturity in Qianhua mutton merino.

No.	Chr.	Chr.ID	Pos	Start(bp)	End(bp)	Position(bp)	P-value	Genes
1	13	NC_040264.1	70195324	70137016	70180747	43731	1.44E-06	RALGAPB
2	4	NC_040255.1	77778627	77695905	77892170	196265	1.71E-06	OSBPL3
3	22	NC_040273.1	50180996	50258669	50267029	8360	2.13E-06	DOCK1
4	10	NC_040261.1	51544225	51282951	51512834	229883	2.38E-06	KLF5
5	5	NC_040256.1	63431663	63239208	63372613	133405	2.39E-06	AFAP1L1
6	5	NC_040256.1	48247772	48131924	48182104	50180	3.40E-06	SEC24A
7	22	NC_040273.1	42450759	42462352	42481845	19493	3.97E-06	FAM45A
8	17	NC_040268.1	33327028	33217243	33247959	30716	4.36E-06	INTU
9	1	NC_040252.1	278241881	278317951	278357928	39977	4.52E-06	RAB6B
10	4	NC_040255.1	64047388	64039868	64080739	40871	4.54E-06	IMMP2L
11	16	NC_040267.1	10417052	10447653	10530167	82514	4.89E-06	MCCC2
12	27	NC_040278.1	106871793	106951323	106960075	8752	5.09E-06	LOC101116968
13	22	NC_040273.1	52580389	52497104	52763200	266096	5.21E-06	MGMT
14	15	NC_040266.1	20274517	20183561	20495049	311488	5.86E-06	DDX10
15	10	NC_040261.1	84497564	84183084	84489812	306728	5.89E-06	STK24
16	2	NC_040253.1	182222722	181988757	182393696	404939	5.92E-06	LRP1B
17	3	NC_040254.1	81040689	81035806	81076706	40900	6.03E-06	LHCGR
18	1	NC_040252.1	71245400	71227262	71253832	26570	6.14E-06	ZNF326
19	4	NC_040255.1	54796053	54773414	54799004	25590	6.27E-06	SLC26A3
20	5	NC_040256.1	76721979	76658023	76789907	131884	6.28E-06	GABRG2
21	12	NC_040263.1	33865029	33507201	34332494	825293	7.70E-06	SMYD3
22	6	NC_040257.1	101034611	100910121	100989030	78909	7.74E-06	LOC101105754
23	7	NC_040258.1	97027136	96951607	97084668	133061	7.95E-06	STON2
24	17	NC_040268.1	62387246	62278441	62309161	30720	8.42E-06	WDR66
25	6	NC_040257.1	127419907	127489169	127577176	88007	8.77E-06	LRPAP1
26	11	NC_040262.1	59709078	59663647	59692986	29339	9.75E-06	C11H17orf67
27	6	NC_040257.1	23094381	23113162	23142629	29467	1.04E-05	GSTCD
28	5	NC_040256.1	42666557	42757863	42758804	941	1.17E-05	LOC101111094
29	5	NC_040256.1	9332692	9282740	9345301	62561	1.18E-05	ADGRE2
30	4	NC_040255.1	126670987	126146659	127048336	901677	1.20E-05	DPP6
31	17	NC_040268.1	9372986	9083290	9421624	338334	1.20E-05	IQCM
32	17	NC_040268.1	63381436	63354372	63381143	26771	1.23E-05	PPTC7
33	7	NC_040258.1	38432101	38491993	38493176	1183	1.24E-05	TMEM62
34	27	NC_040278.1	6124539	6109953	6390877	280924	1.26E-05	ANOS1
35	4	NC_040255.1	52586993	52574143	52629243	55100	1.27E-05	PUS7

36	24	NC_040275.1	39559968	39437955	39558440	120485	1.35E-05	RNF216
37	8	NC_040259.1	98007330	98013330	98160964	147634	1.35E-05	WDR27
38	7	NC_040258.1	14283659	14191242	14264741	73499	1.37E-05	ZWILCH
39	17	NC_040268.1	72916913	72938067	72977676	39609	1.43E-05	KCTD10
40	26	NC_040277.1	47979435	47674038	48041660	367622	1.43E-05	ZNF385D
41	6	NC_040257.1	92614612	92577430	92641932	64502	1.44E-05	LOC114115323
42	19	NC_040270.1	39645055	39612004	39906475	294471	1.46E-05	SYNPR
43	5	NC_040256.1	57634755	57689595	58004278	314683	1.47E-05	YIPF5
44	17	NC_040268.1	74902731	74756771	74999262	242491	1.58E-05	MYO18B
45	25	NC_040276.1	12108309	11945497	12346437	400940	1.59E-05	CHRM3
46	13	NC_040264.1	67604042	67680835	67700378	19543	1.61E-05	LOC101120595
47	17	NC_040268.1	18319934	18180123	18466395	286272	1.66E-05	RNF150
48	25	NC_040276.1	15717133	15449276	15830608	381332	1.75E-05	ANK3
49	14	NC_040265.1	14009570	13991048	13998057	7009	1.87E-05	KLHDC4
50	17	NC_040268.1	78828026	78839019	78846040	7021	1.88E-05	TCN2
51	14	NC_040265.1	7636156	7577965	7744742	166777	1.89E-05	CDYL2
52	24	NC_040275.1	40626622	40306251	41023191	716940	1.92E-05	SDK1
53	23	NC_040274.1	62879133	62769751	62827945	58194	2.04E-05	FECH
54	8	NC_040259.1	53870472	53860832	53879267	18435	2.04E-05	GABRR1
55	22	NC_040273.1	38018324	38081375	38319474	238099	2.06E-05	AFAP1L2
56	22	NC_040273.1	48989574	48896997	49288779	391782	2.10E-05	ADAM12
57	22	NC_040273.1	6392336	5545522	6592088	1046566	2.13E-05	PCDH15
58	27	NC_040278.1	142882624	142960383	143010334	49951	2.14E-05	LOC101122504
59	26	NC_040277.1	18622355	18648099	18654695	6596	2.24E-05	TRIML2
60	24	NC_040275.1	29884118	29870512	30297340	426828	2.25E-05	GALNT17
61	22	NC_040273.1	38208737	38081375	38319474	238099	2.33E-05	ABLIM1
62	25	NC_040276.1	15657118	15653843	15654159	316	2.44E-05	LOC101120721
63	-	NW_020997908.1	6736200	6406862	6950542	543680	2.45E-05	LOC114112505
64	20	NC_040271.1	30896706	30889402	30902278	12876	2.46E-05	MOG
65	20	NC_040271.1	34767286	34736079	34736474	395	2.47E-05	SCGN
66	24	NC_040275.1	29582941	29311310	29832077	520767	2.70E-05	CALN1
67	24	NC_040275.1	21776028	21839409	21867850	28441	2.73E-05	COG7
68	11	NC_040262.1	34623605	34535996	34560499	24503	2.94E-05	STX8
69	20	NC_040271.1	42571849	42522972	42600068	77096	3.01E-05	RNF144B
70	22	NC_040273.1	36227241	36116321	36129569	13248	3.01E-05	VTI1A
71	24	NC_040275.1	7762855	7762337	7776724	14387	3.02E-05	METTL22
72	24	NC_040275.1	12001709	11857135	12207646	350511	3.11E-05	SHISA9
73	24	NC_040275.1	40042129	40026714	40079969	53255	3.45E-05	FOXK1
74	24	NC_040275.1	20804464	20857368	20929749	72381	3.58E-05	METTL9
