# Supplementary material for: Genome-wide association research on the reproductive traits of Qianhua Mutton Merino sheep
Source: Anim Biosci. 2024 Apr 1;37(9):1535–47. doi: 10.5713/ab.23.0365 (PMC11366534; doi:10.5713/ab.23.0365)
Supplement: Supplementary file 6 [file ab-23-0365-Supplementary-Table-6.pdf]

Table S6. Results of the genome-wide association analysis of weaning weight in Qianhua mutton merino.

No.	Chr.	Chr.ID	Pos	Start(bp)	End(bp)	Position(bp)	P-value	Genes
1	27	NC_040278.1	12107634	12087063	12093217	6154	4.88E-07	LOC101121916
2	27	NC_040278.1	116125468	116056245	116690109	633864	1.39E-06	TENM1
3	27	NC_040278.1	132230972	132220852	132348137	127285	1.59E-06	PAK3
4	16	NC_040267.1	27617184	27461532	27632591	171059	2.05E-06	PELO
5	27	NC_040278.1	3703785	3642421	4042458	400037	3.06E-06	NLGN4X
6	27	NC_040278.1	14540281	14496258	14542137	45879	4.42E-06	CLTRN
7	27	NC_040278.1	63727224	63471751	63961524	489773	4.43E-06	OPHN1
8	27	NC_040278.1	139912536	139881889	140462673	580784	4.80E-06	IL1RAPL2
9	11	NC_040262.1	44412624	44375414	44535378	159964	4.87E-06	LOC101117683
10	26	NC_040277.1	33611955	33414208	34162646	748438	6.21E-06	UNC5D
11	11	NC_040262.1	50125239	50147667	50347913	200246	7.05E-06	ACACA
12	15	NC_040266.1	35045572	34886835	35052444	165609	8.21E-06	SORL1
13	27	NC_040278.1	24463311	24264836	24559435	294599	8.41E-06	POLA1
14	19	NC_040270.1	17279578	17259747	17296699	36952	9.48E-06	TCAIM
15	27	NC_040278.1	33198543	32486721	34591843	2105122	9.73E-06	DMD
16	27	NC_040278.1	107771649	107610224	107674554	64330	1.08E-05	SLC9A6
17	27	NC_040278.1	18501505	18489952	18605098	115146	1.79E-05	MAP3K15
18	18	NC_040269.1	4593660	4061932	4887636	825704	1.79E-05	GABRG3
19	10	NC_040261.1	56559515	56354027	56625125	271098	1.97E-05	MYCBP2
20	14	NC_040265.1	7578334	7524802	7534948	10146	2.08E-05	CDYL2
21	20	NC_040271.1	48819564	48783570	48839831	56261	2.18E-05	SYCP2L
