# Supplementary material for: Genome-wide association research on the reproductive traits of Qianhua Mutton Merino sheep
Source: Anim Biosci. 2024 Apr 1;37(9):1535–47. doi: 10.5713/ab.23.0365 (PMC11366534; doi:10.5713/ab.23.0365)
Supplement: Supplementary file 7 [file ab-23-0365-Supplementary-Table-7.pdf]

**Table S7.** Results of the genome-wide association analysis of daily weight gain before weaning in Qianhua mutton merino.

| No. | Chr. | Chr.ID | Pos | Start(bp) | End(bp) | Position(bp) | P-value | Genes |
|---|---|---|---|---|---|---|---|---|
| 1 | 25 | NC_040276.1 | 34726127 | 34723522 | 34843932 | 120410 | 3.22E-05 | DLG5 |
| 2 | 11 | NC_040262.1 | 23715917 | 23736554 | 23740451 | 3897 | 1.15E-06 | LASP1 |
| 3 | 1 | NC_040252.1 | 211029106 | 211010165 | 211057936 | 47771 | 2.85E-06 | TMEM44 |
| 4 | 27 | NC_040278.1 | 116125468 | 116030731 | 116051386 | 20655 | 3.17E-06 | TENM1 |
| 5 | 19 | NC_040270.1 | 19762607 | 19724632 | 20269763 | 545131 | 4.07E-06 | GRM7 |
| 6 | 12 | NC_040263.1 | 68051580 | 68118337 | 68184868 | 66531 | 4.65E-06 | GLUL |
| 7 | 27 | NC_040278.1 | 12107634 | 12105420 | 12167112 | 61692 | 5.11E-06 | LOC101121916 |
| 8 | 1 | NC_040252.1 | 70786698 | 70844280 | 70927105 | 82825 | 5.35E-06 | LRRC8B |
| 9 | 11 | NC_040262.1 | 6747051 | 6660726 | 6662760 | 2034 | 1.03E-05 | FADS6 |
| 10 | 10 | NC_040261.1 | 25157234 | 25070584 | 25116804 | 46220 | 1.04E-05 | LHFPL6 |
| 11 | 27 | NC_040278.1 | 14540281 | 14578847 | 14583687 | 4840 | 1.28E-05 | CLTRN |
| 12 | 6 | NC_040257.1 | 102328865 | 102244499 | 102361121 | 116622 | 1.42E-05 | CNOT6L |
| 13 | 27 | NC_040278.1 | 139912536 | 139881889 | 140462673 | 580784 | 1.48E-05 | IL1RAPL2 |
| 14 | 23 | NC_040274.1 | 67555453 | 67515370 | 67709168 | 193798 | 1.84E-05 | BCL2 |
| 15 | 24 | NC_040275.1 | 35678712 | 35381744 | 35732647 | 350903 | 2.04E-05 | CUX1 |
| 16 | 20 | NC_040271.1 | 7999928 | 7765484 | 8063092 | 297608 | 2.06E-05 | MLIP |
| 17 | 11 | NC_040262.1 | 44412252 | 44375414 | 44535378 | 159964 | 2.67E-05 | LOC101117683 |
| 18 | - | NW_020997233.1 | 15114 | 3847 | 18614 | 14767 | 1.08E-04 | LOC114112283 |
| 19 | 11 | NC_040262.1 | 989196 | 758851 | 1071683 | 312832 | 1.25E-05 | CEP112 |
| 20 | 25 | NC_040276.1 | 32497292 | 32271931 | 33415964 | 1144033 | 1.67E-05 | LRMDA |
| 21 | 19 | NC_040270.1 | 48582600 | 47928893 | 48806868 | 877975 | 1.91E-05 | CACNA2D3 |
